# Leucine-rich alpha 2 glycoprotein is a new marker for active disease of tuberculosis

**DOI:** 10.1038/s41598-020-60450-3

**Published:** 2020-02-25

**Authors:** Minoru Fujimoto, Tomoshige Matsumoto, Satoshi Serada, Yusuke Tsujimura, Shoji Hashimoto, Yasuhiro Yasutomi, Tetsuji Naka

**Affiliations:** 1 Department of Clinical Immunology, Kochi Medical School, Kochi University, Nankoku, 783-8505 Japan; 2 Department of Medicine, Osaka Prefectural Hospital Organization Osaka Habikino Medical Center, Habikino, 583-8588 Japan; 3 Department of Internal Medicine, Osaka Anti-Tuberculosis Association Osaka Hospital, Neyagawa, 572-0854 Japan; 4 Laboratory of Immunoregulation and Vaccine Research, Tsukuba Primate Research Center, National Institutes of Biomedical Innovation, Health and Nutrition, Tsukuba, 305-0843 Japan; 5 Department of Clinical Research Center, Osaka Prefectural Hospital Organization Osaka Habikino Medical Center, Habikino, 583-8588 Japan; 6 Laboratory of Immune Signal, National Institutes of Biomedical Innovation, Health and Nutrition, Ibaraki, 567-0085 Japan

**Keywords:** Diagnostic markers, Tuberculosis

## Abstract

Tuberculosis (TB) caused by *Mycobacterium tuberculosis* (Mtb) is a global health problem. At present, prior exposure to Mtb can be determined by blood-based interferon-gamma release assay (IGRA), but active TB is not always detectable by blood tests such as CRP and ESR. This study was undertaken to investigate whether leucine-rich alpha-2 glycoprotein (LRG), a new inflammatory biomarker, could be used to assess active disease of TB. Cynomolgus macaques pretreated with or without Bacille Calmette-Guerin (BCG) vaccination were inoculated with Mtb to induce active TB. Blood was collected over time from these animals and levels of LRG as well as CRP and ESR were quantified. In the macaques without BCG vaccination, Mtb inoculation caused extensive TB and significantly increased plasma CRP and LRG levels, but not ESR. In the macaques with BCG vaccination, whereas Mtb challenge caused pulmonary TB, only LRG levels were significantly elevated. By immunohistochemical analysis of the lung, LRG was visualized in epithelioid cells and giant cells of the granulation tissue. In humans, serum LRG levels in TB patients were significantly higher than those in healthy controls and declined one month after anti-tubercular therapy. These findings suggest that LRG is a promising biomarker when performed following IGRA for the detection of active TB.

## Introduction

Tuberculosis (TB) is a symptomatic disease caused by replicating *Mycobacterium tuberculosis* (Mtb). According to the WHO global tuberculosis report 2018 (https://www.who.int/tb/publications/global_report/en/), about 23% of the world’s population is estimated to be latently infected with Mtb, Of these, up to 10% of humans develop active disease after Mtb infection^1^, which makes TB a major global health problem with the estimation of 10.4 million new cases and 1.7 million deaths in 2016^2^. Importantly, 40% of estimated TB cases remain to be undiagnosed^2^. Rapid detection of active TB is critical to reduce TB-related death.

Recently-introduced interferon gamma release assay (IGRA) has better specificity than tuberculin skin test (TST) in TB screening and now become the standard for the identification of Mtb sensitization^1^. Nevertheless, IGRA does not differentiate between active TB and resolved/latent infection^3^. At present, sputum smear or culture of Mtb is still a gold standard for diagnosis of active TB. In addition, sputum culture is also a major biomarker for the evaluation of treatment^1,4,5^. However, sputum test is assessable only when sputa of good quality were obtained from patients. Accordingly, sputum test is unavailable for infants with TB and patients with extrapulmonary tuberculosis. In addition, sputum test is not always available in resource-poor settings. Moreover, the sputum culture is not suitable for rapid decision making during therapy of outpatients, given that it requires long time before obtaining results^1^. To detect Mtb in sputa in a rapid turnaround time, molecular diagnostics such as the GeneXpert MTB/RIF assay may be applicable^5^. Nevertheless, this assay is expensive and does not discriminate live and dead bacteria. Therefore, convenient biomarkers are urgently needed for detecting active TB and/or evaluating treatment response.

One feasible strategy to detect active TB is the measurement of acute phase proteins (APPs). APPs are produced primarily in the liver in response to inflammatory cytokine stimulation. Although APP elevation is not specific to TB, it is widely used as a serological marker for various infectious diseases. The most representative marker is serum C-reactive protein (CRP) that is also increased in TB patients. However, while CRP has some benefits for diagnosis of advanced TB^6,7^, it likely has a limitation in detecting less severe cases^8^.

In order to develop a new inflammatory biomarker, we previously took advantage of proteomics technology and identified leucine-rich α2 glycoprotein (LRG) as a marker for evaluating disease activity of rheumatoid arthritis and other immune diseases^9^. Like other APPs, LRG is synthesized in hepatocytes and its production is upregulated in response to systemic inflammation^10^. Interestingly, however, LRG production is detectable not only in hepatocytes but also in other cell types including neutrophils, macrophages and epithelial cells in inflamed tissues^11^. Moreover, LRG induction is dependent not only on IL-6, but also on other inflammatory cytokines such as IL-1β, IL-22 and TNF-α. These characteristics are probably the advantage of LRG over CRP, given that CRP is produced primarily in the liver only in the presence of IL-6. Accordingly, LRG was better than CRP in evaluating the mucosal healing of patients with ulcerative colitis^12^ and the disease activity of rheumatoid arthritis patients during therapy with IL-6 blockade^13^. Given that there has been no report about the utilization of LRG in detecting active TB, here we examined LRG levels in a non-human primate (NPH) model of TB and in human TB patients.

## Results

**Figure 1:**
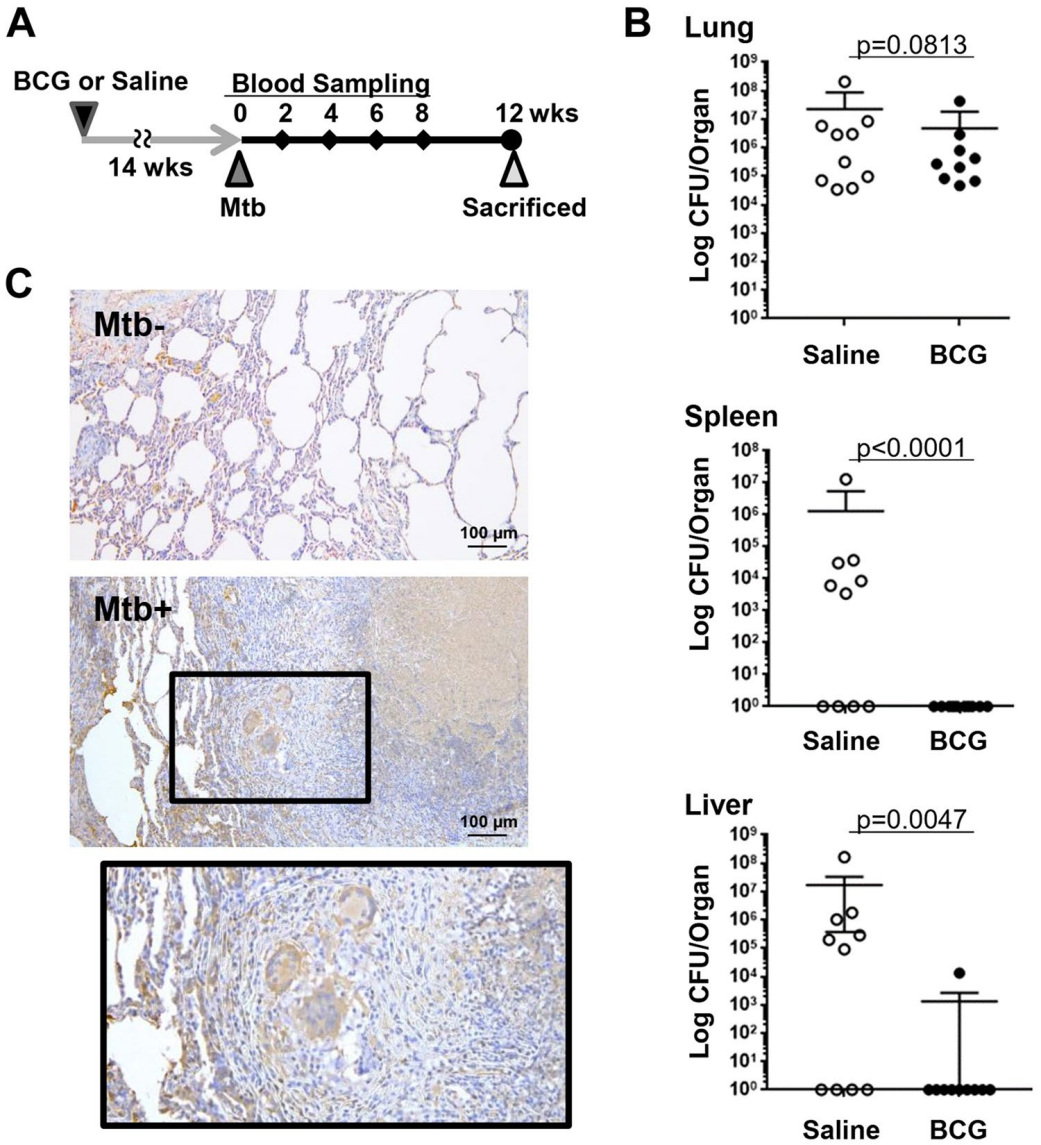
A non-human primate model of tuberculosis. (**A**) Cynomolgus macaques were pre-treated with saline (n = 10) or BCG (n = 10) and challenged by Mtb. After the collection of blood samples at 0, 2, 4, 6, 8 weeks after Mtb challenge, monkeys were sacrificed for analysis at 12 weeks. (**B**) Bacterial burden of organs in macaques with or without BCG pretreatment at 12 weeks after Mtb challenge. Indicated organs were harvested 12 weeks after Mtb infection. Live Mtb counts (CFU) in organs were determined by the mycobacterial culture of organ homogenates. Bars represent mean ± SEM. *p < 0.05, by Mann-Whitney’s U test. (**C**) Immunohistochemical analysis of LRG. Paraffin sections of lung tissues obtained from Mtb-unchallenged (Mtb−) and Mtb-challenged (Mtb+) macaques were used for immunohistochemical analysis to visualize LRG protein expression in tuberculosis lesions.

The immune response of non-human primate (NHP) to Mtb is known to be similar to that of humans^14^. To evaluate the potential usefulness of LRG as a marker for active TB, we used a NHP model of TB in this study. Cynomolgus macaques were pretreated with Bacille Calmette-Guerin (BCG) vaccination (n = 10) or with saline (n = 10) and then were infected intratracheally with Mtb (Fig. 1A). Evaluation of live bacteria in organs indicated that Mtb infection was established in these macaques (Fig. 1B). Notably, whereas BCG vaccination failed to prevent Mtb infection in the lung, it could significantly reduce dissemination of Mtb to extrapulmonary organs such as the liver and spleen.

We next examined the expression of LRG protein in lesions of pulmonary TB. As shown in Fig. 1C, increased LRG protein was visualized in Langerhans giant cells, epithelioid cells and alveolar cells of the tuberculosis lesion, suggesting that LRG production is upregulated locally in the lung after Mtb infection.

**Figure 2:**
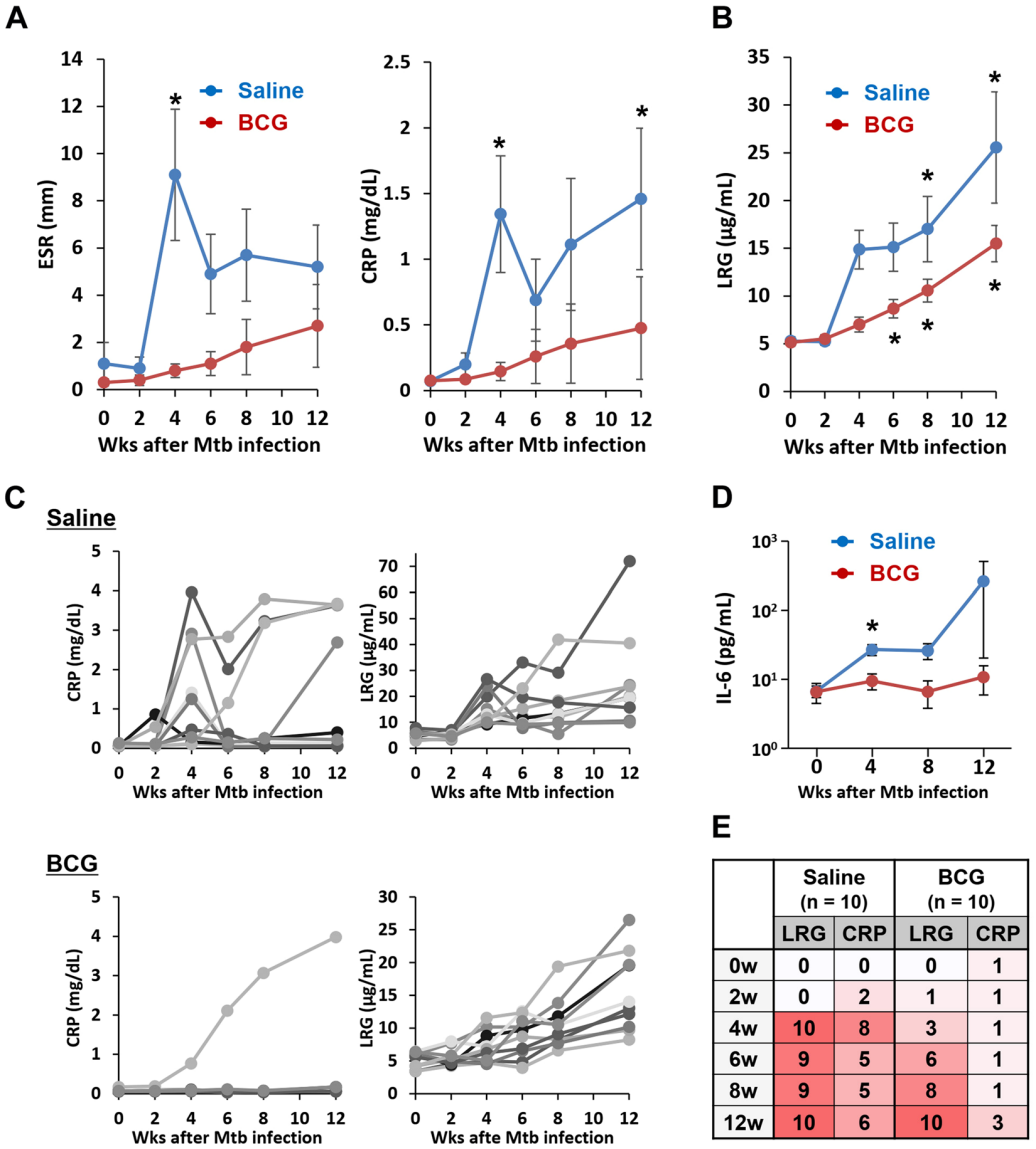
Time-course analysis of inflammatory biomarkers in macaques with Mtb infection. (**A**,**B**) Mean values of biomarkers for the saline-treated and BCG-treated groups. Blood samples were harvested from cynomolgus macaques challenged by Mtb as indicated in Fig. 1A (n = 10 each for the saline group and the BCG group). Levels of ESR and CRP in these macaques were determined by laboratory tests (**A**). LRG levels were determined by ELISA (B). Data were shown as mean ± SEM. *p < 0.05 by a Sidak test. (**C**) Values of biomarkers for each macaque. Laboratory data of each monkey from the saline group (n = 10) and the BCG group (n = 10) was shown separately. (**D**) IL-6 levels in plasma collected at 0, 4, 8, 12 weeks after Mtb challenge. Plasma IL-6 levels were determined by a bead-based immunoassay. Data were shown as mean ± SEM. *p < 0.01 by Scheffe’s test. (**E**) Number of TB macaques with elevated levels of LRG or CRP. Cutoff values of LRG (7.80 μg/mL) and CRP (0.147 mg/dL) were defined as the mean + 1.96 SD of uninfected cynomolgus macaques.

We then measured levels of erythrocyte sedimentation rate (ESR), plasma CRP and plasma LRG in these macaques at 0, 2, 4, 6, 8 and 12 weeks after infection. As shown in Fig. 2A, levels of ESR and CRP in saline-treated macaques significantly increased at 4 weeks after Mtb infection. Nevertheless, ESR levels declined thereafter and only CRP remained to be significantly high at 12 weeks after infection. In contrast, LRG levels in saline-treated monkeys gradually increased and significant elevation of LRG was observed at 8 and 12 weeks after infection (Fig. 2B). In comparison to the saline-treated group, levels of biomarkers in the BCG-treated group were lower, in accordance with less severe disease in the latter group. Of note, ESR and CRP in BCG-treated monkeys showed no significant increase after Mtb infection (Fig. 2A). In fact, clear elevation of CRP (>0.5 mg/dL) was observed only in one out of 10 BCG-treated monkeys (Fig. 2C), in accordance with the poor elevation of systemic IL-6 in these monkeys compared to saline-treated animals (Fig. 2D). In sharp contrast, LRG levels in the BCG-treated group were increased gradually in all macaques (Fig. 2C) and significantly elevated at 6, 8 and 12 weeks after infection (Fig. 2B). When the higher limits of normal values for CRP and LRG were estimated as mean + 1.96 SD of uninfected monkeys, LRG levels at 12 weeks were elevated in all monkeys irrespective of BCG vaccination (Fig. 2E), but CRP elevation, if any, was observed only in 4 out of 10 monkeys with BCG vaccination.

**Figure 3:**
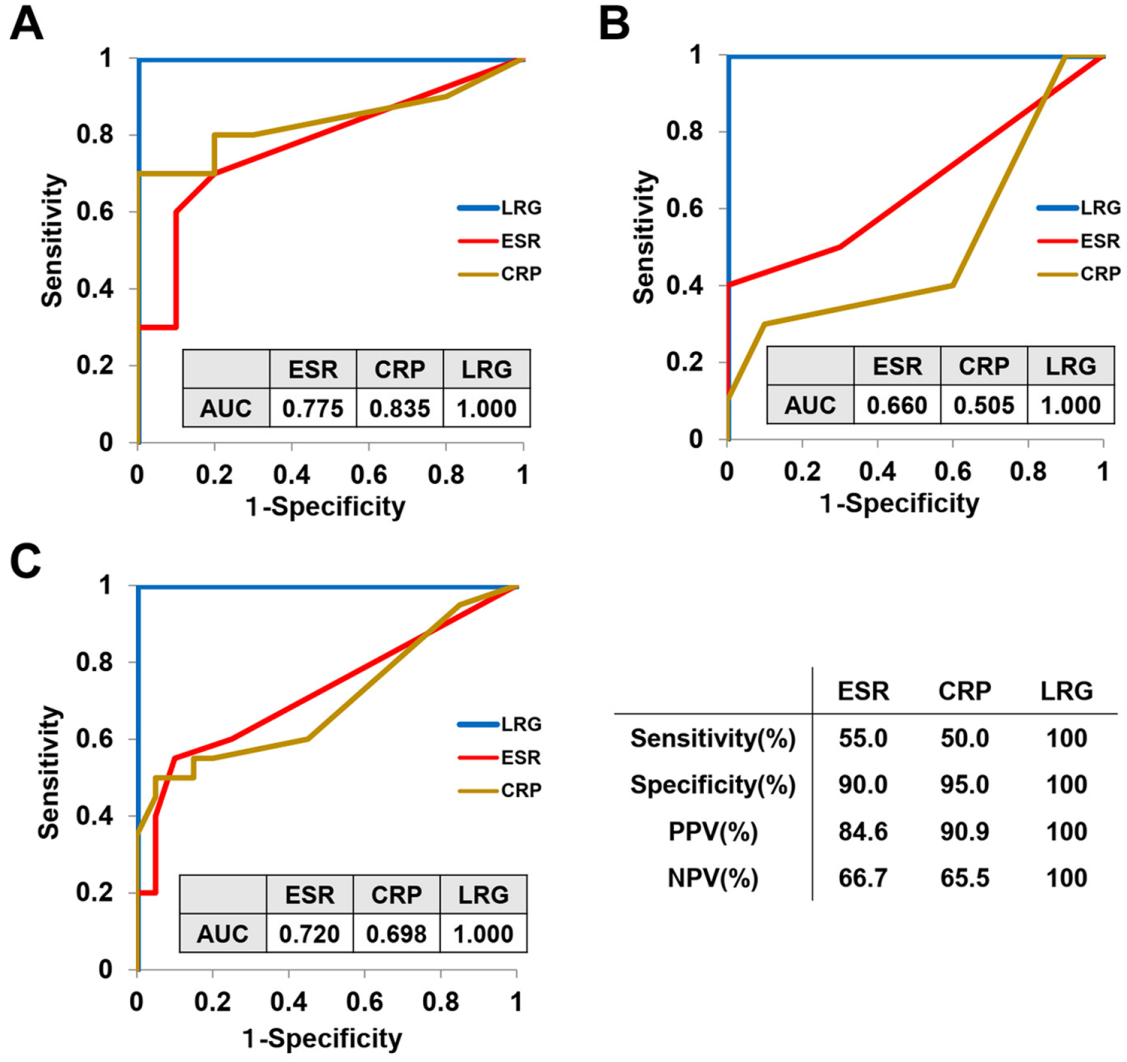
Diagnostic performance of inflammatory biomarkers for active tuberculosis in macaques. Receiver operating characteristic (ROC) curve analyses for ESR, CRP and LRG in discrimination of active TB at 12 weeks after Mtb challenge were performed on the saline-treated macaques (**A**), BCG-treated macaques (**B**) and all macaques (**C**). The value of area under the ROC curve (AUC) of each biomarker is shown in the figure. Optimal cutoff values for ESR (2 mm/hr), CRP (0.14 mg/dL) and LRG (8.22 μg/mL) were determined by the ROC curve analysis of all macaques (**C**) and were used to calculate sensitivity, specificity, positive predictive value and negative predictive value of each biomarker.

To compare the diagnostic performance of inflammatory biomarkers in detecting active TB at 12 weeks after Mtb challenge, the receiver operating characteristic (ROC) curves were generated. The area under the curve (AUC) for LRG was higher than those for ESR and CRP in saline-treated macaques (Fig. 3A). Moreover, AUC for LRG in BCG-treated animals was dramatically better those for ESR and CRP (Fig. 3B). Similar AUCs were obtained when all macaques were analyzed together (Fig. 3C). When the optimal cutoff values were determined by ROC curve analysis of all macaques (Fig. 3C), the sensitivity, specificity, positive predictive value and negative predictive value for LRG in discriminating macaque TB were 100%, 100%, 100% and 100%, respectively, which were superior to those for ESR (55.0%, 90.0%, 84.6% and 66.7%, respectively) or those for CRP (50.0%, 95.0%, 90.9% and 65.5%, respectively). These results suggest that LRG is a better marker than ESR or CRP in detecting active TB, particularly for those that received BCG vaccination.

**Figure 4:**
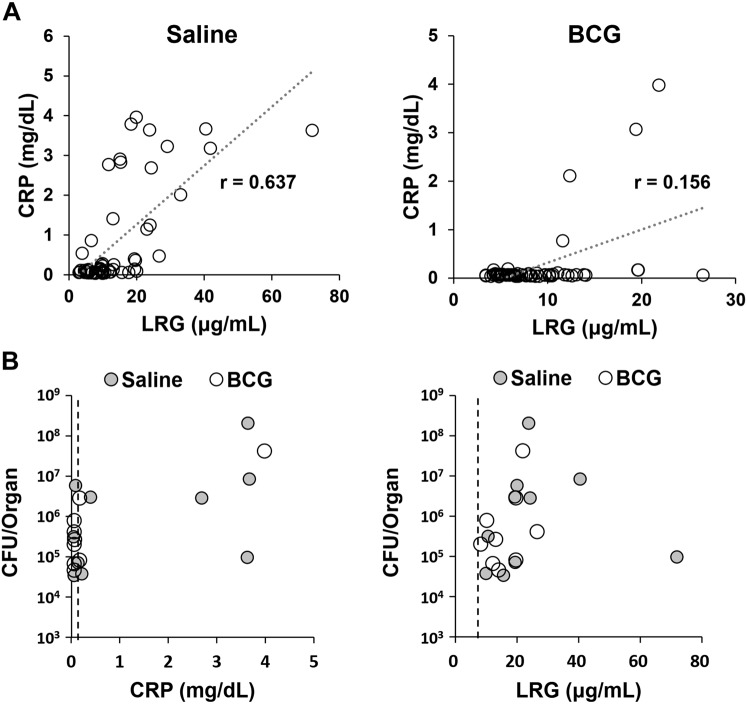
Comparison of CRP and LRG levels in macaques with Mtb infection. (**A**) Correlation analysis of CRP and LRG in macaques with TB. Data of the saline group and the BCG group were shown separately. Each graph contains 60 data from 10 animals (Blood samples harvested 6 times per each macaque). Correlation was assessed by Spearman’s correlation analysis. (**B**) Levels of plasma CRP or LRG are depicted with bacterial load in the lung. Dotted lines indicate cutoff values for CRP (0.147 mg/dL) and LRG (7.80 μg/mL).

We next examined the correlation between CRP and LRG in macaques during the course of Mtb infection. In saline-treated monkeys, CRP considerably correlated with LRG (r = 0.637, Fig. 4A). On the contrary, in BCG-treated monkeys, CRP remained low in most of animals and did not correlate with LRG (r = 0.156, Fig. 4A), We also assessed the association between biomarkers and the severity of pulmonary TB. As shown in Fig. 4B, plasma LRG levels, in comparison to those of CRP, tended to increase in parallel with mycobacterial burden in the lung, suggesting that LRG roughly reflects active inflammation caused by live Mtb.

**Figure 5:**
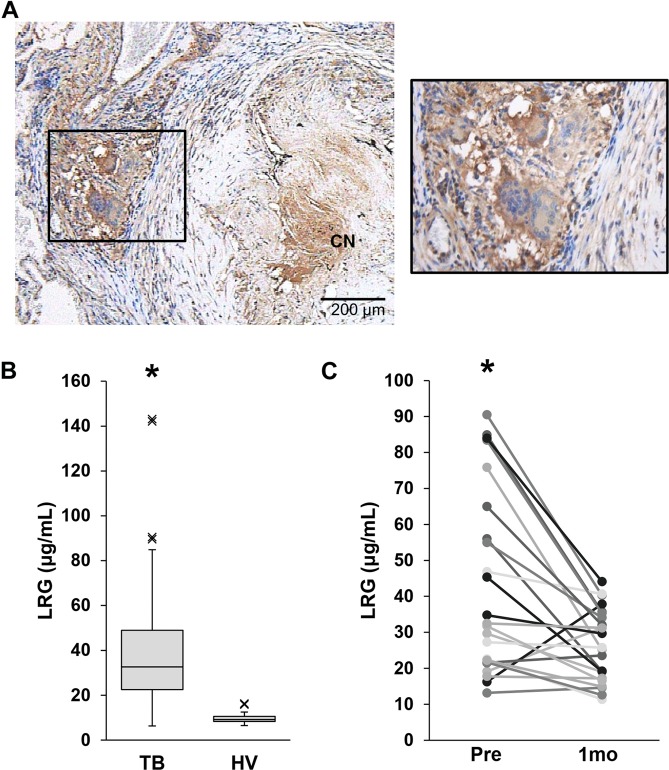
LRG levels in the lung tissue and sera of patients with tuberculosis. (**A**) Localization of LRG in the lung lesion of a patient with tuberculosis. Paraffin section of the lung was obtained from a patient with TB. LRG was visualized by immunohistochemical staining. CN (caseous necrosis). Bar = 200 μm (**B**) Serum LRG levels in tuberculosis patients (n = 86) and healthy volunteers (n = 31) were determined by ELISA. Data are presented as Tukey’s boxplots (median, upper and lower quartiles). *p < 0.001, by t-test. Blood samples were obtained from patients (n = 23) with active tuberculosis before and 1 month after starting a standard anti-tuberculosis therapy. Levels of LRG in these TB patients were determined by ELISA. *p < 0.001, by paired t-test.

We then examined whether LRG is useful for the evaluation of TB in humans. We first evaluated the expression of LRG in pulmonary lesion of TB by immunohistological staining of the lung from a TB patient. As shown in Fig. 5A, strong signal was obtained in the granulation tissue adjacent to caseous necrotic center. Closer examination demonstrated the expression of LRG in the cytoplasm of Langhans’ giant cells and epithelioid cells (Fig. 5A). We next compared serum levels of LRG in TB patients with healthy volunteers. As shown in Fig. 5B, LRG levels in TB patients were significantly higher than those in healthy volunteers, indicating that LRG is a good marker for active TB. We then examined the time course of LRG before and after antitubercular drug treatment. As depicted in Fig. 5C, LRG levels were significantly decreased one month after the initiation of antitubercular drugs. These results suggest that LRG is a biomarker that can evaluate the effectiveness of antitubercular therapies.

## Discussion

In the present study, we used the NHP model of TB. NHP is considered to be the best animal model of tuberculosis because, due to the evolutional relationship of NHP with humans, their immune response to Mtb closely resembles that of humans^14,15^. In addition, like humans, acute phase reactants including not only LRG but also CRP are elevated in NHP during inflammation. Using this model, we first showed that LRG is a better marker than CRP and ESR in detecting active TB, in particular of previously BCG vaccinated macaques. In addition, in macaques with TB, protein levels of LRG were increased in the pulmonary lesion, suggesting that LRG can reflect local inflammatory response. We then showed that serum LRG is elevated in humans with active TB and is declined promptly one month after therapy. These results suggest that LRG can be a marker for active TB in humans and also a measure for the effect of anti-tubercular treatment.

This study indicates that CRP and ESR in macaques are not suitable as markers for active TB, in particular when animals had received BCG vaccination. BCG vaccination is universally recommended in many countries and is expected to reduce severe TB patients in humans^16^. However, it compromises the diagnostic value of TST^17^ due to the antigenic overlap between BCG and TST. Moreover, our study raises the possibility that BCG vaccination might also compromise the diagnostic performance of conventional inflammatory biomarkers, by down-modulating acute phase response. Notably, the diagnostic performance of CRP in TB has been a matter of debate in Japan^8^ where BCG vaccination has been given to most babies. In particular, in children with TB, traditional markers including CRP and ESR were often not elevated^18^. In this study, LRG levels in all TB monkeys were increased from the baseline, even in those vaccinated with BCG. Our data suggest that LRG may serve as a better marker for active TB than CRP and ESR in the countries where most people have been vaccinated with BCG.

It should be noted that LRG is potentially a biomarker for a variety of diseases and its elevation in blood is not specific to TB. We originally identified LRG as an inflammatory biomarker for autoimmune diseases such as rheumatoid arthritis and inflammatory bowel disease (IBD)^9^. Subsequent studies from our group and others have shown that LRG is increased in various immune-related diseases such as adult-onset Still’s disease^19^, psoriasis^20^, juvenile idiopathic arthritis^21^, Kawasaki disease^22^, appendicitis^23^ and cancers^24,25^, indicating that LRG elevation is not limited to autoimmune diseases. In addition, LRG may serve as a biomarker for several other disease conditions such as heart failure^26^, diabetes-related complications^27–29^ and obesity^30^, which are not typical inflammatory diseases but may involve immunopathogenic mechanisms. Thus, for diagnosis of active TB, LRG should be used in conjunction with other laboratory tests such as IGRA. Recently, we have developed an LRG quantitative kit together with a company. This kit is commercially available in Japan and is compatible with automatic analyzers of clinical laboratories. So far, LRG has been approved in Japan as a marker of disease activity in IBD. In future, LRG may also be applicable to other diseases including TB.

It is of interest to examine why BCG vaccination disrupted the correlation between LRG and CRP in Mtb-infected macaques. To gain insight into the mechanism of this phenomenon, we measured IL-6 and several cytokine/chemokine levels (IL-10, IP-10, IL-1β, IL-12 p40, IL-17A, IFN-β, IL-23, TNF-α, IFN-γ, GM-CSF, IL-8, MCP-1; data not shown) in plasma of monkeys before and after Mtb inoculation. In saline-pretreated macaques that developed disease in multiple organs, levels of IL-6 as well as several other factors such as IP-10, IL-17A, IL-23, GM-CSF and MCP-1 were increased significantly after Mtb infection (data not shown). In contrast, in macaques pretreated with BCG vaccination, none of these cytokines/chemokines were increased significantly in plasma (data not shown). Whereas systemic elevation of IL-6 is required for the induction of CRP in hepatocytes, LRG induction is mediated by multiple inflammatory cytokines and is observed not only in the liver but also in the inflamed tissues such as tuberculosis lesions of the lung. Thus, in BCG-treated monkeys in which systemic cytokine/chemokine elevation was undetectable, immune response localized to lung lesions may be responsible for local LRG expression, leading to a mild but significant increase in the circulating LRG. In accordance with this, plasma LRG in BCG-treated macaques tended to increase as their bacterial burdens increased in the lung. Similarly, LRG levels tended to be high in TB patients with sputum smear 2+ to 3+, although the difference from those with smear results that were 1+ or negative was not significant (data not shown). Further studies are required to clarify the detailed mechanism of LRG induction in TB lesions.

Our findings on macaques and humans suggest that LRG is a promising biomarker for detecting active TB when used in combination with other TB tests such as IGRA. However, this study was done by analysing limited number of TB patients and requires validation in larger cohort studies. In particular, given that all patients in this study attained sputum conversion, we need to examine whether LRG may help to identify patients with drug-resistant tuberculosis. It is also of importance to examine whether LRG can detect the onset of active TB in patients with latent TB infection or with acquired immune deficiency syndrome. Despite these limitations, our data provide evidence that LRG is an objective, rapid test that would be more useful than CRP for clinical decision making for TB. In addition, LRG would be applicable to evaluate new agents and/or vaccines for TB in preclinical studies with macaques or in human clinical trials. Further studies are warranted to utilize LRG in clinical practice of TB.

## Methods

### Experimental animals

Cynomolgus macaques (*Macaca fascicularis*) obtained from Indonesia, Philippines and Malaysia have been maintained as closed colony monkeys in indoor facilities of Tsukuba Primate Research Center (TPRC) of National Institute of Biomedical Innovation, Health and Nutrition (NIBIOHN). Several microorganisms, including bacteria, parasites and viruses, have been eliminated from these monkeys to establish a specific pathogen-free (SPF) colony^31^. All animals were negative for *Mycobacterium spp*., *Shigella, Salmonella*, simian immunodeficiency virus, simian type D retrovirus, simian T-cell lymphotropic virus, simian foamy virus, Epstein-Barr virus, cytomegalovirus and B virus. Twenty healthy male cynomolgus macaques from the breeding colony were stratified into two groups (saline and BCG) of ten animals each. Randomization was conducted based on their age and body weight (mean age in years [range]: saline = 5.0 [4.0–5.8]; BCG = 5.0 [4.1–6.6], mean weight in kg [range]: saline = 3.6 [2.8–4.6]; BCG = 3.7 [2.9–4.3]). Animal experiments were performed in TPRC, NIBIOHN in accordance with the Guidelines for Animal Use and Experimentation to minimize animal pain and suffering, as set out by the National Institutes of Biomedical Innovation, Health and Nutrition. The protocol was approved by the Animal Welfare and Animal Care Committee of the National Institutes of Biomedical Innovation, Health and Nutrition (Permit Number: DS23-8R2).

### Animal model of tuberculosis

To induce active TB, cynomolgus macaques under anesthesia (7.5 mg/kg ketamine HCL and 3 mg/kg xylazine) were administered intratracheally with 44 CFU *M. tuberculosis* (Erdman strain) in a biosafety cabinet at 14 weeks after BCG (Tokyo strain) or saline intradermal injection. BCG vaccination (2 × 10^6^ CFU/100 μl) were performed in accordance with the manufacturer’s instructions for human use. Chest CT analysis was routinely conducted after Mtb challenge to confirm development of active pulmonary TB. Mtb-challenged monkeys were sacrificed for analysis at 12 weeks post infection. Animal procedures were performed in animal biosafety level 2 (BCG immunization phase) or level 3 (Mtb challenge phase) facilities at TPRC throughout the study.

### Erythrocyte sedimentation rate (ESR)

Whole blood (1.6 ml) was collected into a 3.8% sodium citrate vacuum blood collection tube (TERUMO), and filled into a capillary tube using an ESR system stand (TERUMO). The blood was settled under the influence of gravity and the number of millimeters of clear plasma present at the top of the capillary after 1 hour (mm/hr) was measured.

### C-reactive protein (CRP)

Whole blood was collected into a blood collection tube (plain, 5 ml) and was left to stand at least 30 min at room temperature prior to centrifugation at 1,200 g for 10 min. The CRP values of serum samples were determined using a calibrated Fuji DRYCHEM system (FUJIFILM).

### Bacterial burden

Bacterial burden was quantified at necropsy. Whole lungs were sampled for the presence of viable Mtb postmortem, and the weights are determined. To collect fragments of the same size from same place of the organs of each animal, a template containing evenly spaced 2-cm square holes was randomly placed over the whole organ. Tissue samples were selected randomly and collected through the template using a biopsy punch instrument (Punch biopsy, 5 mm, Kaijirushi). The weights of all samples were determined. Tissue samples were dissociated, serially diluted in distilled water, 10-fold serial dilutions were plated in duplicate on 7H10 agar plates, incubated at 37 °C, and CFUs were counted 3 and 5 weeks later.

### Plasma IL-6 levels

Plasma IL-6 levels in macaques were determined by NHP Inflammation Panel (Biolegend, San Diego, CA, USA) according to the manufacturer’s instructions.

### Participants

The patients presented with symptoms suggestive of TB, such as persistent cough, fever, hemoptysis and weight loss, at Osaka Habikino Medical Center were examined for TB infection using chest X-ray (CXR), IGRA and sputum test following clinical evaluation by physicians. Patients with highly possible TB were recruited but only patients with sputum culture positive for Mtb were included in this study. Patients with comorbid conditions (e.g. interstitial pneumonia, cancer, pyoderma, human immunodeficiency virus infection) that may potentially increase serum LRG levels were excluded from the analysis. As a result, 86 patients (51 males and 35 females; a median age 69.5 [range 25–98] years) were analyzed as TB. Among them, 23 patients agreed repeated measurements of serum LRG concentration during anti-tuberculosis therapy. Paraffin sections of lung granulomas for immunohistochemistry were obtained from resected lung specimen of a patient with active TB. Clinical samples obtained from patients were processed in biosafety level (BSL)-3 facility. Healthy volunteers, comprising 18 males and 13 females with a median age of 32 [range, 23–43] years, were recruited at the National Institute of Biomedical Innovation, Health and Nutrition.

### Ethics statement

Animal experiments were performed in TPRC, NIBIOHN, after approval by the Animal Welfare and Animal Care Committee of NIBIOHN (approval number: DS23-8R2) in accordance with the guidelines for animal experiments at NIBIOHN. The human study was approved by the Ethics Committee of the Osaka Habikino Medical Center (approval number: 773-1), the Ethics Committee of NIBIOHN (approval number: 42-1) and the Ethical Review Board of Kochi Medical School (approval number: 29–97). All patients gave their written informed consent prior to inclusion. The human study was carried out in accordance with the Declaration of Helsinki.

### Quantification of LRG

By an ELISA system using an antibody pair of huLRB0091 and rbLRB0048^13^, levels of macaque plasma LRG and human serum LRG were determined by generating standard curves with recombinant macaque LRG and human LRG, respectively.

### Immunohistochemistry

Immunohistochemistry was performed using the ChemMate Envision method (DakoCytomation, Glostrup, Denmark). Briefly, paraffin sections were de-waxed, rehydrated and incubated for 20 minutes in citrate buffer (10 mM citric acid, pH 6.0) at 95 °C–100 °C for antigen retrieval. Sections were treated with 0.3% H_2_O_2_, then blocked with Blocking One (Nacalai, Kyoto, Japan) and incubated with anti-LRG1 polyclonal antibody (HPA001888, 1: 1000; Atlas Antibodies, Stockholm, Sweden) overnight at 4 °C. After washing, sections were treated with Dako ChemMate ENVISION Kit (K5007) according to manufacturer’s instructions. All sections were counterstained with hematoxylin.

### Time course of changes of serum LRG concentration during anti-Tuberculosis medication

We measured serum LRG protein in patients with active tuberculosis and in normal volunteers. We also collected human serum samples from the 33 patients with tuberculosis before and one month after starting standard TB therapies.

### Statistics

For comparisons among two groups, the values were analyzed by Mann–Whitney U-test. For comparisons among repeated measurements of biomarkers, the values were analyzed by one-way ANOVA followed by a Sidak test or Friedman’s test followed by Scheffe’s test. Receiver operating characteristic (ROC) curves were generated to compare the diagnostic performance of biomarkers. Correlation between two parameters were assessed by Spearman’s correlation analysis. The software BellCurve for Excel (Social Survey Research Information, Tokyo, Japan) or Prism (GraphPad Software, San Diego, CA) was used. P values less than 0.05 were considered significant.
